# Animal hoarding cases in England: Implications for public health services

**DOI:** 10.3389/fpubh.2022.899378

**Published:** 2022-08-30

**Authors:** Justine Wilkinson, Mariyana Schoultz, Helen M. King, Nick Neave, Catherine Bailey

**Affiliations:** ^1^Department of Engineering & Environment, Northumbria University, Newcastle upon Tyne, United Kingdom; ^2^Department of Nursing, Midwifery and Health, Northumbria University, Newcastle upon Tyne, United Kingdom; ^3^Hoarding Research Group, Northumbria University, Newcastle upon Tyne, United Kingdom

**Keywords:** animal hoarding, hoarding disorder, public health services, self-neglect, public health nuisance, spatial distribution

## Abstract

Hoarding disorder is characterized by an accumulation of possessions due to excessive acquisition of or difficulty discarding possessions, regardless of their actual value and is estimated to affect 2–6% of the population. Animal hoarding, a distinct subset of hoarding disorder, has a significant public health impact on the humans involved, as well as animal welfare. Individuals exhibit self-neglect, apathy, social withdrawal and object hoarding; living within squalid, deteriorated, structurally unsafe and uninhabitable premises, alongside neglected animals. Cases are complex, costly and impact on a range of responding service providers. Effective case management is poorly understood and researched, with published literature in England particularly sparse. Improving understanding of the characteristics of these cases is the first step in informed case management. This research is the first exploration of the characteristics of animal hoarders in England and the areas where cases were located. Information about prosecutions involving large numbers of animals that were reported in the media was systematically obtained. This identified 66 cases between January 2015 and December 2020. Geospatial analysis exploring characteristics of locations where animal hoarding cases are also reported. Findings were broadly consistent with the international literature in that females (64%), those living alone (71%) and those with a mean age of 49 were well represented. Cats (61.5%) and dogs (60%) were the most commonly hoarded species. There was a mean of 44 animals per case and dead or animals requiring euthanasia found in 53% of cases. Key characteristics of the areas where cases were found highlight urban, densely populated, and high levels of deprivation being the most represented. Evidence of recidivism was evident in 39% of cases, suggesting that prosecution is not an effective rehabilitator. Animal hoarding raises serious implications for Public Health Services, and the lack of current effective case management strategies are discussed.

## Introduction

Until recently, the ownership of large numbers of animals was regarded as an eccentric lifestyle choice, but individuals working in the veterinary, public health and behavioral sciences have raised awareness of this behavior, and its negative effects on personal/public health (1). A definition of animal hoarding is: “*having more than a typical number of companion animals; failing to provide minimum standards of nutrition, sanitation and veterinary care; denial of the inability to provide this minimum care and the impact of that failure on the animals, household and human occupants of the dwelling and persistence, despite this failing in accumulating and controlling animals”* (1–3). The Diagnostic and Statistical Manual of Mental Disorders supports the diagnostic criteria considering animal hoarding as a special manifestation of hoarding disorder, with particularly unhealthy living conditions and poor insight capacity of the individuals (4). Researchers have noted the similarities between animal hoarding and object hoarding (5), and different “types” of animal hoarding have been proposed, namely the “overwhelmed caregiver,” “the rescuer,” “the exploiter” (3), “incipient hoarders” and “breeder hoarders” (6). Previous studies have found that the stereotype of an animal hoarder is an older female, living alone with many cats (7, 8). Whilst this generalization is to some extent supported within the literature, the reality is more nuanced in that males, younger people, those living in multi occupancy dwellings also featured in the literature; and whilst any species of animal can be hoarded (1, 2, 6–13), the literature notes particularly cats and dogs (2, 6–12, 14). Globally there is limited academic research in this field, with published cases being reported in North America (1, 2, 7, 8, 14); Australia (6, 9–11) Spain (12) and Brazil (14, 15). At present there is minimal information regarding animal hoarding in England, one study has reported on the problem of multi-cat households in the North West of England (16) another has reported on use of motivational interviewing in cases of equine hoarding (17) and researchers have raised awareness of animal hoarding and its social/societal consequences, and of its unique challenges for professionals encountering it (1, 7, 14, 18, 19).

The aim of this study was to explore the characteristics of animal hoarders in England. As is typical in other countries (1, 10), England has no single agency responsible for monitoring and addressing animal hoarding. There is no centralized reporting mechanism for such cases, so accurate recording to understand the scale of the problem is poorly understood and documented. Those cases that arise are likely to be underreported due to the secretive and isolated characteristics of the individuals involved (9, 15). To address this, we examined published media case studies involving prosecutions for offenses related to animal hoarding, and in addition used geospatial analysis to explore characteristics of locations where animal hoarding cases were reported. Cases included were those meeting the widely accepted definition of animal hoarding, i.e., an accumulation of a large number of animals and a failure to provide minimal standards of nutrition, sanitation and veterinary care and to act on the deteriorating condition of the animals (including disease, starvation or death) and the negative impact of the hoarding on their own health and wellbeing and that of other household members (1–4); that occurred in England and were reported in the media between 1 January 2015 and 31 December 2020. Large numbers of animals were determined as being >5. Broadcast media such as television and radio were excluded as were cases from Scotland, Wales and Northern Ireland. While this technique is not a new methodology, it is novel in this field, with only one other known paper comparing frequency and spatial distribution of animal and object hoarding behaviors in Brazil (15). By doing so we hope to be able to compare these reports with those cases reported internationally and identify resulting similarities/differences.

## Methods

**Figure 1:**
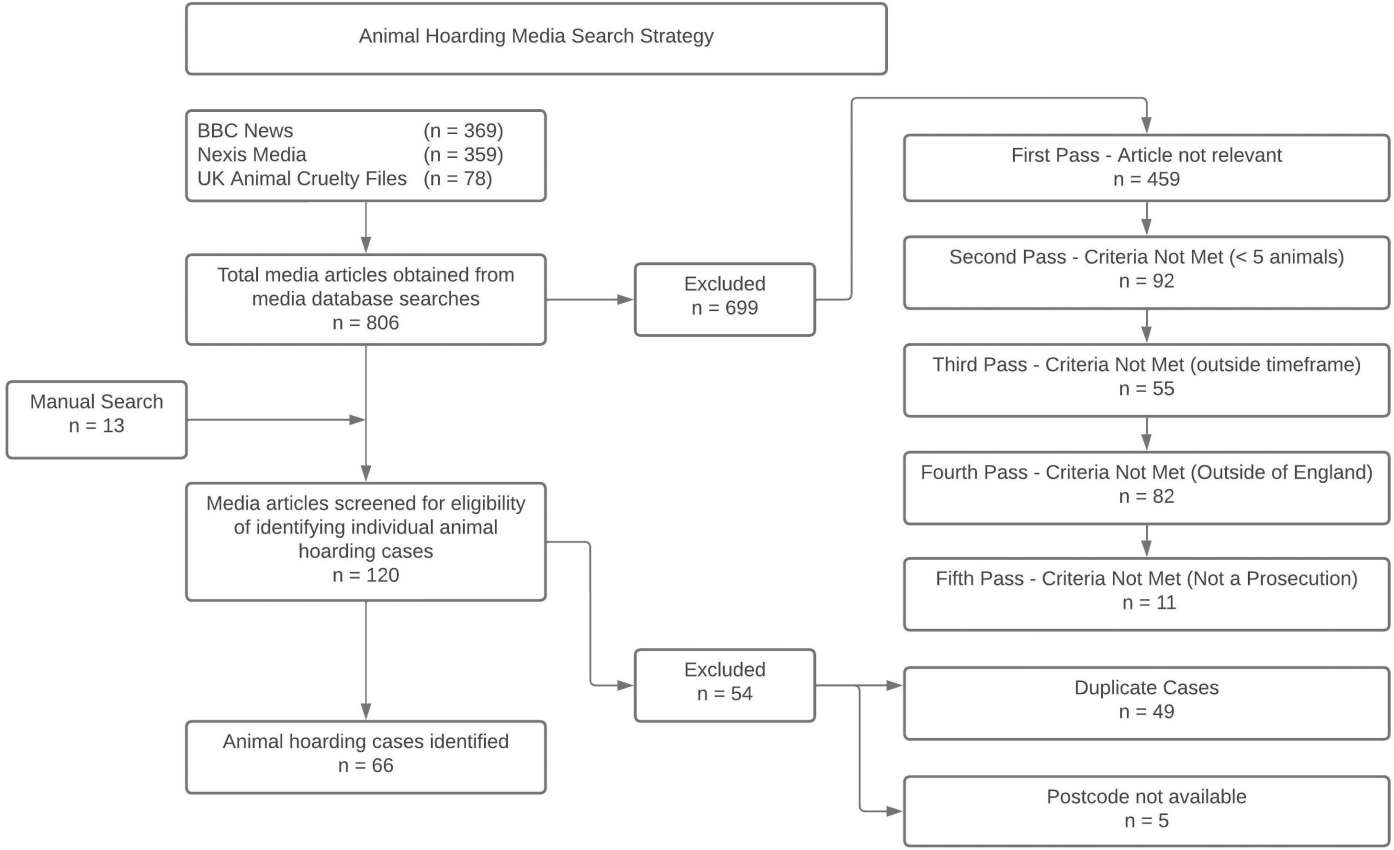
Animal hoarding media search strategy.

Access to case reports undertaken by the leading animal welfare organization with an investigatory section was denied because of concerns that individuals could be identified due to the uniqueness of cases. Therefore, publicly available information from media articles reporting prosecutions involving large numbers of animals were obtained. Reports were sourced from a systematic search strategy of electronic media databases including BBC News, Nexis and UK Animal Cruelty Files using keywords such as “animal ban,” “animal hoard,” “animal squalor,” “animal prosecution” which were then screened for meeting the animal hoarding criteria (1). Figure 1 summarizes the search strategy.

A convenience sample of 66 cases reported in local and national media were identified between 1 January 2015 to 31 December 2020. Features of the reports including individual demographics, animal context, services involved, legal actions and case outcomes were recorded. The reports provided personal identifiable data in the public domain including the address, although as an ethical consideration each case was anonymised by use of a unique identifiable reference and case location restricted to postcode only. In cases involving charges against more than one individual (*n* = 18) the characteristics of each person were recorded, for example demographic information, charges and outcomes to avoid double counting.

Exploration of the characteristics of case location by neighborhood was undertaken by geocoding postcode locations by Lower Super Output Areas (LSOAs) using Ordnance Survey lookup tables (20) and by the centroid location of the Postcode unit (~15 addresses). LSOAs are a standard statistical geography designed to be of a similar population size, with an average of approximately 1,500 residents or 650 households (21), and are commonly used for spatial analysis of neighborhoods, council resource allocation and for research purposes (20, 22). Following a review of animal hoarding literature (2, 8–10, 12, 15) selected NOMIS datasets (23) (Table 1), were explored to assess the characteristics of case locations relating to key basic demographic information associated with the population living in a LSOA and the characteristics of that location such as rural/urban and nature of housing stock and tenure. To allow comparisons, English Indices of Multiple Deprivation (IMD) (24, 25), at LSOA level, which closely covered the data collection period, provided a measure of relative deprivation based on income, employment, education, skills and training, health and disability, crime, barriers to housing and services and the living environment. NOMIS datasets for each LSOA (not already categorized, such as IMD 2015 and 2019) were assigned a decile and then ranked. The number of cases by decile and/or category for each dataset were reviewed to identify clusters of cases. For example, were there a higher number of cases found in rural or urban categories, or in LSOAs with a high or low deprivation?

**Table 1:** Summary of NOMIS datasets (23) selected to explore the characteristics of animal hoarding case locations.

**Category**	**Dataset**	**Extracted information (all as of Census Day 27 March 2011 except for indices of multiple deprivation)**
Population	Age structure and LSOA (KS102EW)	Estimated age structure, mean and median ages of usual residents of England and Wales aged 59 and under and over 60
	Population density (QS102EW)	Estimated population density (number of persons per hectare) for usual resident population of England and Wales
	General health and LSOA (KS301EW)	Estimated self-assessed state of health (Very good, Good, Fair, Bad and Very bad) for usual residents in England and Wales
Area	Accommodation type – households and LSOA (QS402EW)	Estimated household accommodation type (Bungalow, detached, semi-detached, terraced including end, flat/ maisonette/ apartment, purpose-built block of flats or tenement, converted or shared household) classification for England and Wales
	Tenure and LSOA KS402EW	Estimated households by tenure owned or rented for England and Wales
	Rural-urban classification (RUC 2011)	Estimated rural-urban classification of output areas. Urban or rural based on its population weighted center being either greater or <10,000 people
Area and population	Indices of multiple deprivation 2015 and 2019	Estimated relative measure of deprivation for small areas in England (ranked from 1 most deprived to 32,844 least deprived). Domains include income, employment, education, skills and training, health and disability, crime, barriers to housing and services and the living environment. Datasets for 2015 and 2019 extracted to compare the time period of the case reports.

Office for National Statistics (23).

Ministry of Housing, Communities & Local Government (24).

Ministry of Housing, Communities & Local Government (25).

## Results

### Descriptive statistics

Sixty-six reports across England involving 83 individuals met the criteria with the gender split being 64% female (n = 53) and 36% male (*n* = 30). In cases where there was more than one person at the property, only one set of hoarding behavior characteristics was counted to avoid duplication, for example number of animals at the property. The mean age for the whole sample was 49 (18–79) years, the female mean age being 48.9, and the male being 48.7. Twenty-seven percent of the sample (*n* = 22) were aged 60 or over. Most cases involved people living alone 71% (*n* = 47). The total number of animals reported, was known in 55 cases as 2,411 with a mean of 44 per case. In 11 cases the total number was estimated as 472 with a mean of 43 per case. As some of the previous literature (1, 2, 6–13), had reported an excess of females in animal hoarding cases, we conducted an initial one-way ANOVA to compare the number of animals hoarded by males and females. This revealed no significant difference (F_1_,_76_ =0.133, *p* = 0.716).

The frequency of the most common animal species hoarded were cats (61.5%), closely followed by dogs (60%), small mammals (19%), birds (including fowl) (17.9%), horses (10.3%), reptiles (12.8%), farm animal (5.1%) and other (12.8%). In 48% (*n* = 32) of cases, more than one animal species was hoarded. Typical cases described people living in squalid conditions with many animals that were alive, sometimes dead or in poor physical condition. Dead animals or animals in such poor condition that were euthanised were found in 53% (*n* = 35) of cases. Table 2 compares our findings with Nadal and colleagues (26) who summarized animal hoarding features from 26 empirical investigations.

**Table 2:** Comparison of features of animal hoarding cases between findings from this study and a recent systematic review of empirical investigations.

	**Gender (%)**	**Mean age**	**Single household (%)**	**Mean number of animals per case**	**Frequency of animal species (%)**
This study	Female 64	49	71	44	Cats: 61.5
				Range 5–201	Dogs: 60
Nadal et al. (26)	Female 74.9	55.6	51.8	64.1	Cats: 65.2
				Range 6–918	Dogs: 61

The most common charges were breaches of the Animal Welfare Act 2006 (27) (95%). The most common outcome was for some or all the animals to be removed in 98% of cases (*n* = 65) this was followed up by disqualification orders in 90% (*n* = 75) of cases. Suspended sentences were issued to 32 individuals with an average sentence of 18.6 weeks (range 6 weeks to 18 months) and a custodial sentence was issued to 14 individuals with an average sentence of 18.7 weeks (range 6 weeks to 2 years). Community Orders including unpaid work were issued to 18 individuals with an average length of 133.6 h (range 50 to 200 h), rehabilitation days were issued to 18 individuals with an average of 25.8 days (range 10 to 60 days) and a curfew was issued to 4 individuals. One case received a conditional discharge. A fine was issued in 23% (*n* = 19) of cases with a mean fine of £371 (range £100–£9,000). There was evidence of recidivism in 39% of cases (*n* = 26) including breach of previous disqualification orders in 20% of cases (*n* = 13). Costs were awarded in 80% (*n* = 66) of cases with a mean award of £1,683 (range £85–£50,00).

### Characteristics of lower-layer super output areas

**Figure 2:**
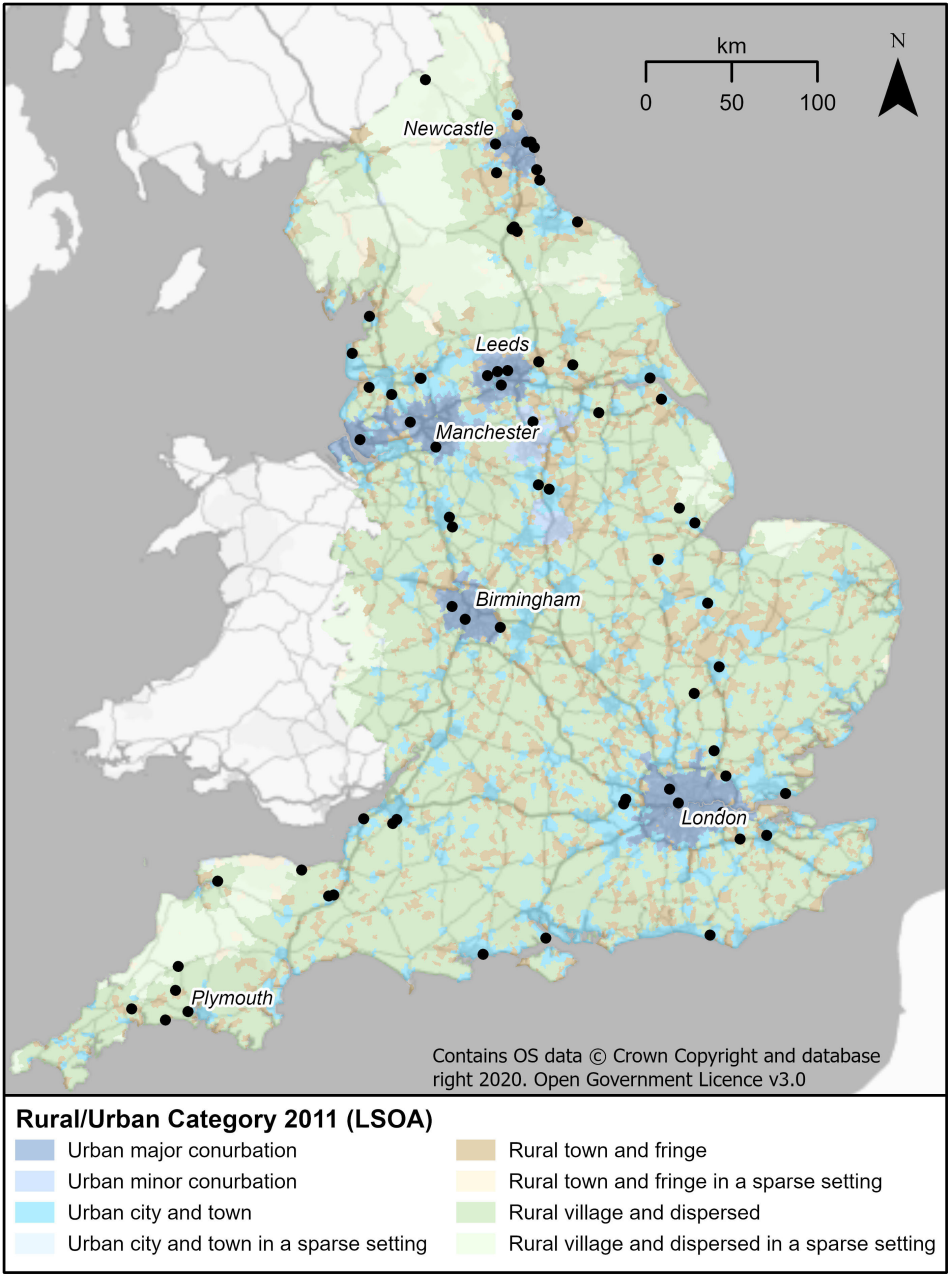
Location of animal hoarding cases.

The spatial distribution of media reported prosecutions by postcode were spread across England (see Figure 2).

Characteristics of the LSOA containing animal hoarders based on decile rank/category suggest whilst all deciles included cases, those that were more densely populated had higher case counts (23 cases in percentiles lower than the 40th decile compared with 42 in the 50th percentile and higher, with the 70th percentile having the highest count). This is based on 65 cases as one postcode could not be identified. Comparison was made between rural and urban areas, based on the Rural-Urban Classification 2011 aggregated at LSOA level. A higher count of cases in those areas classified as Urban, i.e., settlements of more than 10,000 people was found. The highest count of cases was found in “Urban city and town” (32/65 cases), followed by “Urban major conurbation” (14/65 cases). Whilst cases did occur in areas classified as rural, they were fewer with “Rural town and fringe” having the highest count (5/8 cases). LSOAs with higher numbers of rented properties had higher numbers of cases of animal hoarders (35 cases in deciles 50 and above compared to 30 in deciles 40 and below). The number of cases counted in areas with the highest deprivation based on IMD 2019 was 47 (comparing a count of 46 for IMD 2015), as opposed to areas with lower deprivation that had case counts based on the IMD 2019 measure of 18 (IMD 2015 measure was 19). Associated with IMD is a self-reported health classification. It was also found that areas with cases of animal hoarders were more likely to be found in areas with “bad” (47/65) or “very bad” (42/65) self-reported general health. The characteristic of age was explored to identify if areas with higher numbers of an older population (over 60) had a higher number of cases. The 50th percentile and above had a higher count of cases in areas with higher representation of over 60,s (count 42) with the 70th percentile having the highest count compared with the 40th percentile and below (count 23).

## Discussion

Historically, animal hoarders have been reported to be older females, living alone, with many cats (1, 9). Our study found that whilst women did form the majority of reported cases (64%) the mean age of 49 was lower than that reported in other studies; and men, those under the age of 60, and those living as family units were also well-represented. Pet ownership is believed to bring physical and psychological benefits to their owners (28, 29) but these are counteracted in a hoarding situation. Boundaries are less likely to be in place for where animals play, eat, sleep, urinate and defecate leading to severely impaired living conditions from cluttered properties that are difficult to clean and maintain, and deteriorated use of functional living spaces (30). The resulting unsanitary, foul smelling, pest infested, squalid conditions present potential risk of disease and nuisance to occupant(s), visitors and neighbors (31–35). Our findings show that companion animals were the most frequently hoarded species, with cats slightly more common than dogs (61.5 vs. 60%). This is consistent with the literature with various studies reporting that either cats or dogs form the most hoarded species (26). It is not clear whether this is a cultural difference associated with ownership ratio, although in the UK dogs are more commonly owned than cats (36). In line with previous research (1, 37) examples of other species being hoarded was found, including small mammals, birds, horses, farm animals and reptiles, but to a lesser extent than cats and dogs. The mean number of animals per case was 44 which was lower than reported by a recent study (26) (64.1) although the range suggests that this figure could be skewed by a small number of large cases (our study ranged from 5 to 201 compared with 6–918) (26). The number of animals present challenges to responding organizations in terms of veterinary services, public health services, ongoing housing and care of animals, property clean up as well as legal costs which are unlikely to be recoverable through the court system. Costs requested were rarely stated in the media reports, although one example illustrating the shortfall was for a request of £290,000 costs with only £50,000 being awarded. The high level of costs involved in these cases is consistent with other countries for example, one study estimates cases as costing tens of thousands of Australian dollars (9).

The descriptions of animal suffering and the failings and denials of defendants to act was the starkest illustration of meeting the criteria of animal hoarding. Reports described the properties as having dead or animals in such poor condition that euthanasia was required in 53% of cases, somewhat higher than reported in other studies (7, 9, 10, 12), and much higher than others (6, 14). This gives a grim description of the deteriorated living conditions for both humans and animals. As these were prosecutions, these cases are likely skewed to illustrate the more extreme consequences of animal hoarding, supporting the need for early intervention. No single service or organization has responsibility for animal hoarding and no statutory or centralized reporting mechanism exists in the UK, a position common with other countries such as North America (37, 38). The incidence of animal hoarding is therefore difficult to accurately determine, but as an initial estimate the RSPCA, the organization most involved in welfare cases, are reported to receive ~1,000 calls annually for multi-household animal welfare concerns (16). This suggests a crude incidence of 1.78 cases per 100,000 (based upon 1,000 calls in 2019, with England's population in 2019 being 56,286, 961) (39) broadly comparable to the reported incidence of object hoarding at 2.5% (40). Animal hoarding is not a phrase commonly used in the media nor by professionals, meaning case identification is difficult, and recorded under different categories such as self-neglect, public health nuisance or animal welfare. The complexity and extreme nature of animal hoarding cases means that it is likely multiple agencies will be involved at some point responding to specific aspects of concern. The main authors' experience is that often multiple agencies are working with the same households without being aware of the others involvement. This situation is made difficult due to confidentiality and data sharing restrictions due to the Data Protection Act (2018) (41).

Animal hoarding is believed to occur in every community but is poorly understood (2). This study found an even distribution of cases across England supporting one view that this is “everyone's concern” (18). The literature describing geographical characteristics is limited, with one study finding that cases were less likely to be in major cities or urban areas (9), but others reporting the opposite (2, 15). This study found more cases were present in urban areas, particularly within the subcategory of “urban city and town.” This subcategory however represents 43.2% of the population so it could just be that there are more people living in these areas, and therefore more likely to find cases (12, 15). A further consideration is that as cases tend to arise based on complaints, it could be that there is less available space in urban areas, and more rental properties so the problem is identified earlier than for more sparsely populated areas, the deteriorating situation being more overt and becoming a problem for others who report to statutory services to intervene. This reinforces the characteristic of the secretive nature of hoarders (9, 15, 37). One study found that animal hoarding cuts across demographic and socio-economic boundaries (2), however, another found that hoarding frequency was inversely proportional to neighborhood income, as neighborhood income decreases the number of identified hoarders increases (15). Our study finds support for this, as while cases were found in neighborhoods across the spectrum, there were more cases in neighborhoods with higher levels of deprivation. There is no known literature where housing tenure as a characteristic of animal hoarding is reported for comparison. Home ownership (owned outright or with a mortgage) is the most common tenure (64%) in England and Wales based on the 2011 census. It is therefore of interest that a characteristic of the neighborhoods was a higher count of rented properties, this could suggest a transient habit of animal hoarders. It was possible for example to track at least one of the cases who moved across multiple boundaries replicating hoarding behaviors, although this theory would need further research.

Animal suffering is unacceptable and places responding organizations in a moral and ethically challenging position. Animal welfare and individual freedoms are a fine balance and no one agency can determine a holistic opinion, for example a vet may be able to determine animal suffering but not human self-neglect. The act of abrupt removal of animals, prosecuting and effectively criminalizing individuals with potential mental health issues also poses questions of public interest. Legal action is only part of the solution with high levels of recidivism (up to 100%) reported (7, 42). This study supports the ineffectiveness of the legal system at rehabilitating defendants, evidence of recidivism was identifiable in 39% of cases, including 13 cases where prior disqualifications banning ownership of animals were breached. This emphasizes the importance of a multidisciplinary approach to sensitively approach these cases to the benefit of both animal and human subjects. By improving organization understanding of the complexity of animal hoarding, its causes, and characteristics it is hoped will lead to better understanding of these challenging cases and development of more effective interventions.

There were limitations to this study. The media reports are retrospective and so information cannot be directly verified with the individuals involved, although photographs and video footage did go some way to visually appreciating the conditions. It is important not to overstate what the data is capable of presenting, as this was a convenience sample, it was not possible to estimate how many cases are investigated but do not result in prosecution. Rather, this provides an initial review of high-profile cases that have resulted in successful prosecutions so are likely skewed toward the more extreme end of the spectrum. It was not possible to determine the extent of other organizations input, particularly those with responsibilities for human health. Issues of human health such as self-neglect were rarely reported, nor were the human consequences of living in these conditions. The ONS datasets (23) are mostly based on England and Wales data whereas the media reports were for England only, so whilst the best available it still represents a dilution of the data. Spatial analysis of where cases are found also lack key information about each specific case, and so this review only provides an overview of the characteristics of the areas in which cases are found. Initial analysis supports key characteristics of areas such as urban areas of high levels of deprivation and rental housing stock, which would benefit from further research. We believe that these limitations are countered by the contribution to the developing body of international evidence in this field and believe it to be the first exploration of cases in England. Further research is planned to explore service engagement, which is particularly relevant due to the high levels of recidivism and the apparent lack of deterrent of court action.

## Data availability statement

The raw data supporting the conclusions of this article will be made available by the authors, without undue reservation.

## Author contributions

JW led the overall study, contributed to the data collection and interpretation, and wrote the manuscript. MS contributed to the study design and manuscript edits. NN and HK contributed to the study design, data analysis and interpretation, and manuscript edits. CB contributed to the study design. All authors read, contributed to the research design, and approved the final manuscript.

## Conflict of interest

The authors declare that the research was conducted in the absence of any commercial or financial relationships that could be construed as a potential conflict of interest.

## Publisher's note

All claims expressed in this article are solely those of the authors and do not necessarily represent those of their affiliated organizations, or those of the publisher, the editors and the reviewers. Any product that may be evaluated in this article, or claim that may be made by its manufacturer, is not guaranteed or endorsed by the publisher.
